# Spinal Cord Compression as a Consequence of Spinal Plasmacytoma in a Patient with Multiple Myeloma: A Case Report

**DOI:** 10.3390/clinpract11010018

**Published:** 2021-02-25

**Authors:** Rishi Jayesh Trivedi

**Affiliations:** Faculty of Medical Sciences, University of Bristol, Bristol BS8 1TH, UK; rt15928@bristol.ac.uk; Tel.: +44-7715-3286-79

**Keywords:** multiple myeloma (MM), metastatic spinal cord compression, spinal decompression, spinal stabilisation

## Abstract

Multiple myeloma (MM) is a B cell malignancy resulting in osteolytic lesions. Pathological fracture of the vertebral body resulting in spinal cord compression is a common complication and accounts for approximately 5% of patients with MM. To date, there are no definitive guidelines for the treatment of spinal cord compression as a consequence of MM. Radiotherapy has frequently been the preferred form of treatment. Some surgeons, however, feel that spinal lesions in multiple myeloma should be treated in the same manner as spinal metastases from solid organs. I report the management of a 46-year-old gentleman with multiple myeloma that had resulted in neural compression in the lumbar and thoracic areas. Initial emergent treatment in this patient consisted of spinal decompression and stabilisation.

## 1. Introduction 

The spine is the most common site for skeletal metastasis, with lesions of the axial skeleton representing roughly 39% of all bony metastases. Breast, prostate, and lung cancers classically represent the most common primary tumours with propensity to metastasize to the bony spine due to its rich vascular supply and the valveless nature of the epidural venous plexus described by Batson [1,2].

The incidence of metastatic spinal cord compression (MSCC) is up to 80 cases per million people each year [3]. This equates to 4000 cases per annum in England and Wales [4]. MSCC maybe a feature of advanced primary cancer particularly in cancers of breast, lung, and prostate, however it could be a presenting complaint in up to 20% of malignancies. Treatment in MSCC usually involves a multidisciplinary approach with use of corticosteroids, radiotherapy, and surgery all playing a role [5]. However, evidence has suggested that only 50% of patients have a positive response [6]. Multiple myeloma is a haematological malignancy that commonly involves the spine. Vertebral collapse and soft tissue extension of tumour into the spinal canal in multiple myeloma may cause neurological deficit and mechanical instability leading to pain and deformity. Although the primary treatment of myeloma is usually oncological, this case illustrates the successful use of surgery, in a patient who presented with neurological deficit. 

## 2. Case Report 

A 46-year-old gentleman presented with increasing back pain and pain in the left hip for six months. This pain was associated with numbness in the left leg. Over the previous two weeks, his symptoms had become intrusive, resulting in an ability to walk only with the aid of crutches. The patient reported of no weight loss and no bowel or bladder dysfunction. 

On examination, there was tenderness in the lower thoracic spine, lumbar spine, and over the iliac crest on the left side. Neurological examination revealed reduced sensation over the left leg from the groin to the foot. Power was reduced in left toe extension and left ankle dorsiflexion to Medical Research Council (MRC) grading of 3/5. On the right side, sensation was reduced over the little toe. Reflexes were bilaterally brisk in the lower limbs, but plantar reflex was normal. 

**Figure 1:**
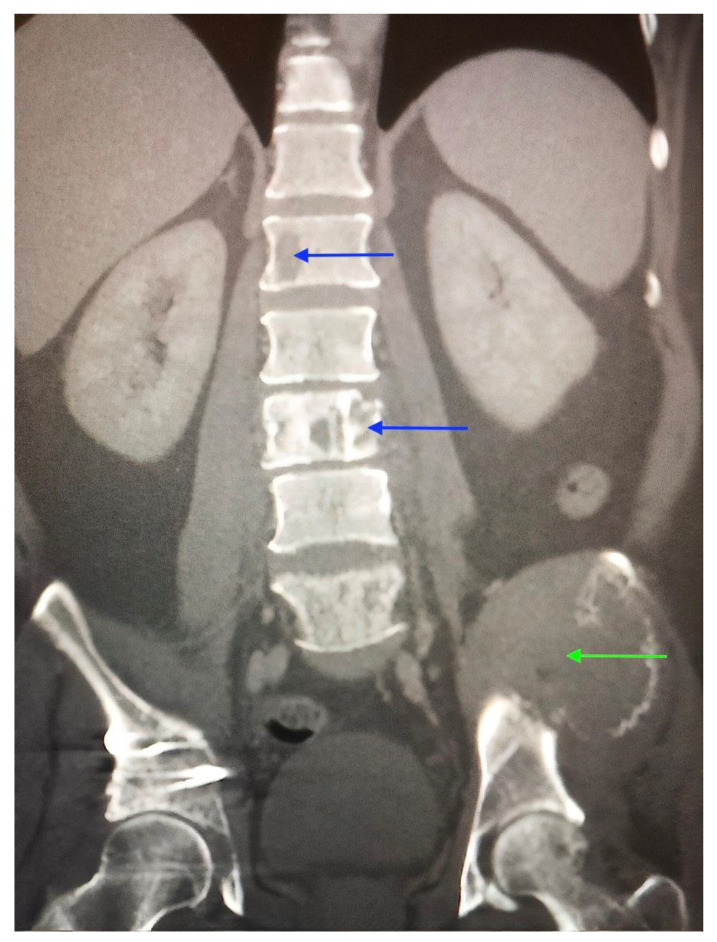
CT scan of the spine and pelvis highlighting multiple areas of bony destruction in the thoracic and lumbar vertebrae. (blue arrows) There is a large mass in the left iliac bone. (green arrow).

**Figure 2:**
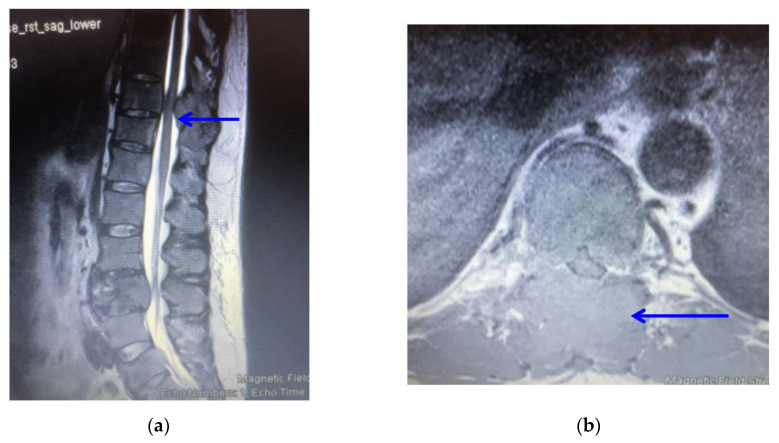
(**a**): T2-weighted sagittal sequence MRI scan showing an epidural soft tissue tumour compressing the spinal cord (blue arrow); (**b**): T2-weighted axial sequence MRI scan at T10 showing marked compression of the spinal cord from a soft tissue tumour (blue arrow).

Full blood count, erythrocyte sedimentation rate (ESR) and calcium levels were normal. Computed tomography (CT) scanning revealed multiple areas of bony destruction in the vertebrae and left iliac bone. (Figure 1) Magnetic resonance imaging (MRI) revealed destruction of T5, T10, L3, and L5 vertebrae with abnormal tissue causing severe compression of the spinal cord and nerves in these areas (Figure 2).

Plasma electrophoresis was performed to check for multiple myeloma. This revealed that IgA and IgM levels were normal. However an abnormal band was detected on plasma electrophoresis. Further analysis revealed an excess of kappa light chains with a value of 200.5 mg/L (normal value 3.3–19.4). Free lambda chain values were normal. These tests suggested a diagnosis of BJP-kappa myeloma.

Tuberculosis can present similarly to MSCC, in that spinal canal involvement can cause radiating pain and limb weakness [7]. It was, however, an unlikely differential diagnosis. The typical manifestations of spinal tuberculosis involve vertebral bone destruction, narrowing of intervertebral disc space and paraspinal abscess [7]. Despite MRI revealing vertebral bone destruction, there was no evidence of paraspinal abscess. 

The possibility of osteomyelitis was also ruled out upon haematological testing. ESR in osteomyelitis tends to be raised to a level greater than 100 mm/h [8]. The patient, however, had a normal ESR and did not present with features of systemic infection.

Primary bone tumours may affect the spine and manifest with cord compression. The incidence of primary bone tumours affecting the spine is reported to be between 4 and 13% [9]. Boussios et al., after review of literature, reported on 69 cases of Ewing’s sarcoma affecting the spine and presenting with cord compression [10]. In their report, the average age of the patient was younger with a median age of 17.95 years. Multiple myeloma is the most common primary bone tumour of the spine. The multiple vertebral involvement in this case would be in favour of either metastatic disease or myeloma.

Lastly, the probability of MSCC was increased by the presence of both motor and sensory symptoms. Radicular pain and sensory complaints tend to be initial symptoms in patients with lumbar metastases, whereas weakness in the limbs is more pronounced in patients with thoracic metastases [11]. As T5, T10, L3, and L5 were all affected, it was concluded that the patient would be treated for spinal cord compression. 

The aim of treatment would not be curative, but rather aimed at improving quality of life [12]. In view of the cord compression, neurological deficit and the mechanical instability caused by vertebral collapse, surgery was proposed as the initial treatment option in this patient. Biopsy was performed from the iliac bone lesion. Biopsy confirmed the diagnosis of myeloma. Histology revealed a highly cellular tumour composed of solid sheets of cells with plasmacytoid morphology. There was a high mitotic rate. Immunohistochemistry revealed a strong expression of CD138 but not for CD56, CD20 or CD3. The proliferation index Ki-67 was approximately 20%.

**Figure 3:**
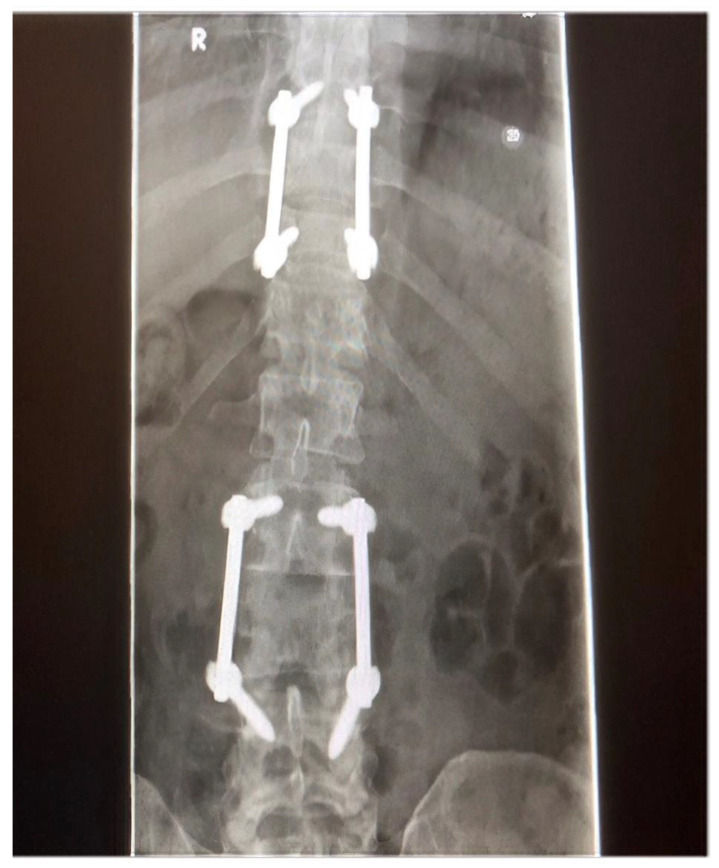
Post-operative x-ray demonstrating spinal stabilisation at the lumbar and thoracic areas using rods and screws.

Subsequently, the patient underwent spinal decompression and spinal stabilisation at the lumbar and thoracic areas. (Figure 3) Histological analysis of surgical specimens was similar to that of the biopsy of the iliac bone confirming a diagnosis of multiple myeloma. Post-operatively radiotherapy was initiated in line with National Institute for Health and Care Excellence (NICE) guidelines [13]. The patient was also referred to the local haematology unit for oncological treatment of the myeloma with the surgical procedure resulting in restoration of mechanical stability to the spine and the surgical decompression offering relief from cord compression and recovery of the neurological deficit.

The operation was successful, with numbness in the left leg improving within 3–4 days. The pain subsided within one month and the patient could walk short distances without the use of crutches. 

At one-year follow-up, the patient regained full function of the spine and hip. Ambulation status was restored, and lower limb power returned to normal (MRC score of 5/5). 

## 3. Discussion 

Spinal cord compression may be the presenting symptom of cancer, as highlighted in this case. A retrospective cohort study reported that 21% of MSCC patients had no pre-existing cancer diagnosis [14]. It may be the presenting feature of advanced primary cancers of lung, breast, and prostate [5]. Low back pain may be the first sign of malignancy; however, if we consider the prevalence of this symptom in our population, it is not unsurprising that the diagnosis of MSCC is often missed. In an observational study of 319 patients with MSCC, a median of two months passed from the onset of pain and the diagnosis of the condition.3 Similarly, in this case, the patient’s back pain was not investigated for six months. It required the onset of motor deficit and limb weakness for red flags to be raised. In the view of this, clinicians should maintain a high index of suspicion for spinal cord compression in a patient presenting with progressive lumbar and thoracic pain. Early detection is pivotal in preserving motor and sensory function. 

This case outlines the efficacy of both MRI and CT scanning in the diagnosis of MSCC. MRI is the gold standard investigation and concurs a sensitivity and specificity of 100% and 93%, respectively [15]. Furthermore, CT scanning, used in conjunction, can aid in preoperative planning, and help detect the site of the primary tumour [16].

Research has showed that surgery for MSCC can provide an improvement in pain, function, and ambulation status. This is in comparison with patients receiving only radiotherapy as treatment for their MSCC [16]. NICE therefore recommends spinal decompression and stabilisation for patients who are deemed fit [17]. Post-operative radiotherapy can be used in conjunction to treat further metastases. The success of this type of treatment was assessed in a prospective randomised control trial. It showed that patients with MSCC treated with direct decompressive surgery plus post-operative radiotherapy retained the ability to walk for longer than patients treated with radiotherapy alone [6]. Surgical treatment further reduced the need for corticosteroids and resulted in increased survival time [6].

Although vertebral involvement is common in multiple myeloma, spinal cord compression occurs in 11–24% of patients [18]. This usually happens from pathological fractures of the affected vertebra. However, compression by extra-osseous soft tissue epidural myeloma is uncommon, being reported in 5% of cases [18]. In this study, compression of the cord at T10 was noted to be from a soft tissue epidural myeloma.

In recent years understanding the patho-biology of myeloma has transformed its treatment, improving survival in patients. The mainstay of treatment remains oncological with chemotherapeutic agents, although use of immunomodulators and monoclonal antibodies is increasingly being employed to improve survival. Despite this, the treatment of patients presenting with cord compression and neurological deficit in multiple myeloma has not been firmly established. Although the role of surgery in patients with MSCC is established, this is less so in multiple myeloma. Kee-Yong Ha et al. presented three cases of multiple myeloma with spinal cord compression [18]. In one patient, radiotherapy and high dose steroid therapy was used. Although there was a resolution of the epidural tumour no neurological recovery occurred. In the second patient, neurological deterioration occurred whilst the patient was being treated with radiotherapy and steroid. Surgical decompression in this patient did not result in any improvement of the neurological deficit. The authors recommended a careful vigil on the neurological status of the patients with early decompression in those with neurological deficit and mechanical instability. In this report the patient had neurological deficit and instability and hence surgical intervention was undertaken prior to pharmacological treatment for the myeloma. Surgical decompression predominantly in solitary myeloma has been reported in other studies [19]. 

Spinal cord injury from compression by tumour may have devastating sequelae with regards to motor function, bowel and bladder control, sensory changes, and sexual function requiring further management strategies [20]. Early intervention is therefore recommended to preserve spinal cord function. 

This report highlights a case of cord compression from a soft tissue epidural tumour in multiple myeloma and the specific use of early surgical intervention to improve neurological function and maintain stability. This resulted in an excellent neurological recovery for the patient. Surgery may play an important role in the treatment of selected patients with multiple myeloma. 

## Data Availability

Data sharing not applicable.
